# ErbB2 inhibition by lapatinib promotes degradation of mutant p53 protein in cancer cells

**DOI:** 10.18632/oncotarget.12878

**Published:** 2016-10-25

**Authors:** Dun Li, Natalia D Marchenko

**Affiliations:** ^1^ Department of Pathology, Stony Brook University, Stony Brook, NY, 11794, USA; ^2^ Department of Pharmacology, Boston University School of Medicine, Boston, MA, 02118, USA

**Keywords:** lapatinib, mutant p53, ErbB2, Hsp90, MDM2

## Abstract

Mutations in the p53 tumor suppressor gene are the most prevalent genetic events in human Her2-positive breast cancer and are associated with poor prognosis and survival. Human clinical data and our *in vitro* and *in vivo* studies strongly suggest potent oncogenic cooperation between mutant p53 and Her2 (ErbB2). Yet, the translational significance of mutant p53 in Her2 positive breast cancer, especially with respect to Her2-targeted therapies, has not been evaluated. Our previous work identified novel oncogenic activity of mutant p53 whereby mutp53 amplifies ErbB2 signaling via the mutp53-HSF1-ErbB2 feed-forward loop. Here we report that pharmacological interception of this circuit by ErbB2 inhibitor lapatinib downregulates mutant p53 *in vitro* and *in vivo*. We found that ErbB2 inhibition by lapatinib inhibits transcription factor HSF1, and its target Hsp90, followed by mutant p53 degradation in MDM2 dependent manner. Thus, our data suggest that mutant p53 sensitizes cancer cells to lapatinib via two complementary mechanisms: mutant p53 mediated amplification of ErbB2 signaling, and simultaneous annihilation of both potent oncogenic drivers, ErbB2 and mutant p53. Hence, our study could provide valuable information for the optimization of therapeutic protocols to achieve superior clinical effects in the treatment of Her2 positive breast cancer.

## INTRODUCTION

Recent evidence suggests that although mutations in the p53 tumor suppressor gene are recognized as “driver” mutations in cancer [1], additional tumor-promoting events, such as cooperation with other oncogenic pathways, are emerging as essential mechanisms of cancer progression [2].

The human epidermal growth factor receptor-2 (Her2, ErbB2) is frequently overexpressed in human breast cancer, which is associated with poor survival [3]. Contrary to Luminal A and B subtypes, sporadic Her2 breast cancer has a high prevalence of p53 mutations (72%) [1] that predict poor prognosis due to a more aggressive disease and increased susceptibility to metastatic recurrence [4]. Furthermore, female patients with germline p53 mutations (Li-Fraumeni syndrome [LFS]) are especially prone to the Her2 subtype of breast cancer (up to 83% of all breast cancer in LFS women [5, 6] compared to 20% in sporadic breast cancer [1]), suggesting cooperative co-selection of these potent oncogenes during Her2 breast cancer progression. This strongly suggests a causative connection between p53 mutations and Her2 breast cancer development. Yet, no systematic studies have been done to assess mutant p53 (mutp53)'s significance in Her2 breast cancer development and therapy.

The main tumor suppressor function of p53 is to respond to cellular stress by activating transcriptional programs that induce apoptosis, growth arrest or senescence. It is widely recognized that when mutated, p53 not only loses its wild-type tumor suppressor functions, but often also actively promotes tumor development by inhibiting wtp53 in a dominant-negative manner or gains novel oncogenic activities, known as gain-of-function (reviewed in [2]). In contrast to the majority of tumor suppressors that are usually inactivated by deletion (i.e. PTEN, Brca1/2, NF1, APC), p53 is typically missense mutated, which suggests a selective advantage of p53 missense mutations over p53 loss. Compared to normal cells, the tight control of mutp53 by MDM2 is diminished in mutp53 tumors, leading to *tumor -specific stabilization* of mutp53, which is thought to be critical for the manifestation of its oncogenic activities (reviewed in [2], [7]). This is strongly supported by *in vivo* studies, e.g. homozygous deletion of Mdm2 in mutp53 knock-in mice leads to further stabilization of mutp53 in tumors and in some normal tissues, shortened tumor latency and enhanced metastases [8]. In support of the oncogenic power of highly stabilized mutp53, we and others have shown that downregulation of mutp53 by RNA interference (RNAi) inhibits the malignant phenotype [9–11]. Knockdown of endogenous mutp53 in human breast (MDA231) and colon cancer (SW480) cells by shp53 suppresses cancer cell growth and invasion *in vitro* and in xenografts [9, 10]. Furthermore, mutp53 downregulation by RNAi decreases cell viability *in vitro* and in xenografts [12], invasion [11, 13] , restores normal mammary architecture in 3D culture in breast cancer cell lines [14], inhibits metastases *in vivo* [15, 16] and suppresses mammary stem cells [17]. Genetic ablation of mutp53 in allotransplanted and autochthonous mouse T/B-lymphoma model curbs tumor growth and extends survival of mutp53 knock-in mice [18]. Together, these proof-of-principle experiments suggest strong addiction to high levels of mutp53 protein in tumors cells. Therefore, depletion of mutp53 in mammary tumors could be therapeutically beneficial. However, pharmacological targeting of mutp53 is a challenging task. Mutp53 is not a surface molecule and does not have enzymatic activity. Hence, identifying the mechanisms of tumor-specific stabilization of mutp53 would open up new therapeutic approaches in the treatment of mutp53 harboring cancer.

Previously we found that compared to p53null counterparts, the mutp53 R172H allele (‘H’ thereafter) aggravates mammary tumorigenesis in the MMTV/ErbB2 mouse breast cancer model, which correlates with amplification of ErbB2 signaling [17]. When dissecting the mechanism of cooperation between ErbB2 and mutp53, we established a novel oncogenic role of mutp53 in the amplification of the ErbB2 pathway *in vivo* and *in vitro* [17, 19]. We found that mutp53 physically interacts with and enhances the transcriptional activity of HSF1 (Heat Shock Transcription Factor 1), the master transcriptional regulator of heat shock proteins (HSP) including Hsp90. In turn, Hsp90 stabilizes its clients ErbB2 and mutp53 itself [19], thereby promoting mammary tumorigenesis [17].

Following this observation, in the present study we demonstrate that the pharmacological interception of the ErbB2-HSF1-mutp53 feed-forward loop by the FDA-approved dual ErbB2/EGFR inhibitor lapatinib destabilizes mutp53 protein in cancer cells. Our data could provide valuable information for the optimization of therapeutic protocols and development predictive biomarkers to achieve superior clinical effects in the treatment of Her2 positive cancer.

## RESULTS

### Lapatinib induces downregulation of mutp53 in ErbB2-expressing mammary cells

Our discovery of the novel oncogenic role of mutp53 in modulation of heat shock response and ErbB2 signaling [17, 19] led us to hypothesize that pharmacological intervention of ErbB2-mutp53-HSF1 loop should diminish HSF1 activity and reduce the levels of its transcriptional target, Hsp90, ultimately leading to destabilization of mutp53, a well-established Hsp90 client [20].

To test this hypothesis we utilized several *in vitro* models: primary cultures of mammary epithelial cells (MECs) and mammary tumors derived from previously described p53−/−;ErbB2 and H/H;ErbB2 mice [17]. To determine whether the observed effects are dependent on the type of p53 mutation, we also established MECs from mutp53 R248Q/-;ErbB2 mice. According to clinical data, codon 248 of the p53 gene is the most frequently mutated in Her2-enriched breast cancer [21]. Thus, we generated a novel breast cancer mouse model by introducing humanized R248Q mutp53 allele [18, 22] (‘Q’ thereafter) into MMTV-ErbB2 transgenic mice. MECs derived from p53 −/−;ErbB2 littermates served as a control. These cell lines, which derived from mice with identical genetic background, provide the unique platform to delineate mutp53-mediated effects in ErbB2 positive cancer. To validate our results in human breast cancer cells we utilized ErbB2 positive human breast cancer cell line BT474 (E285K p53 mutation).

**Figure 1:**
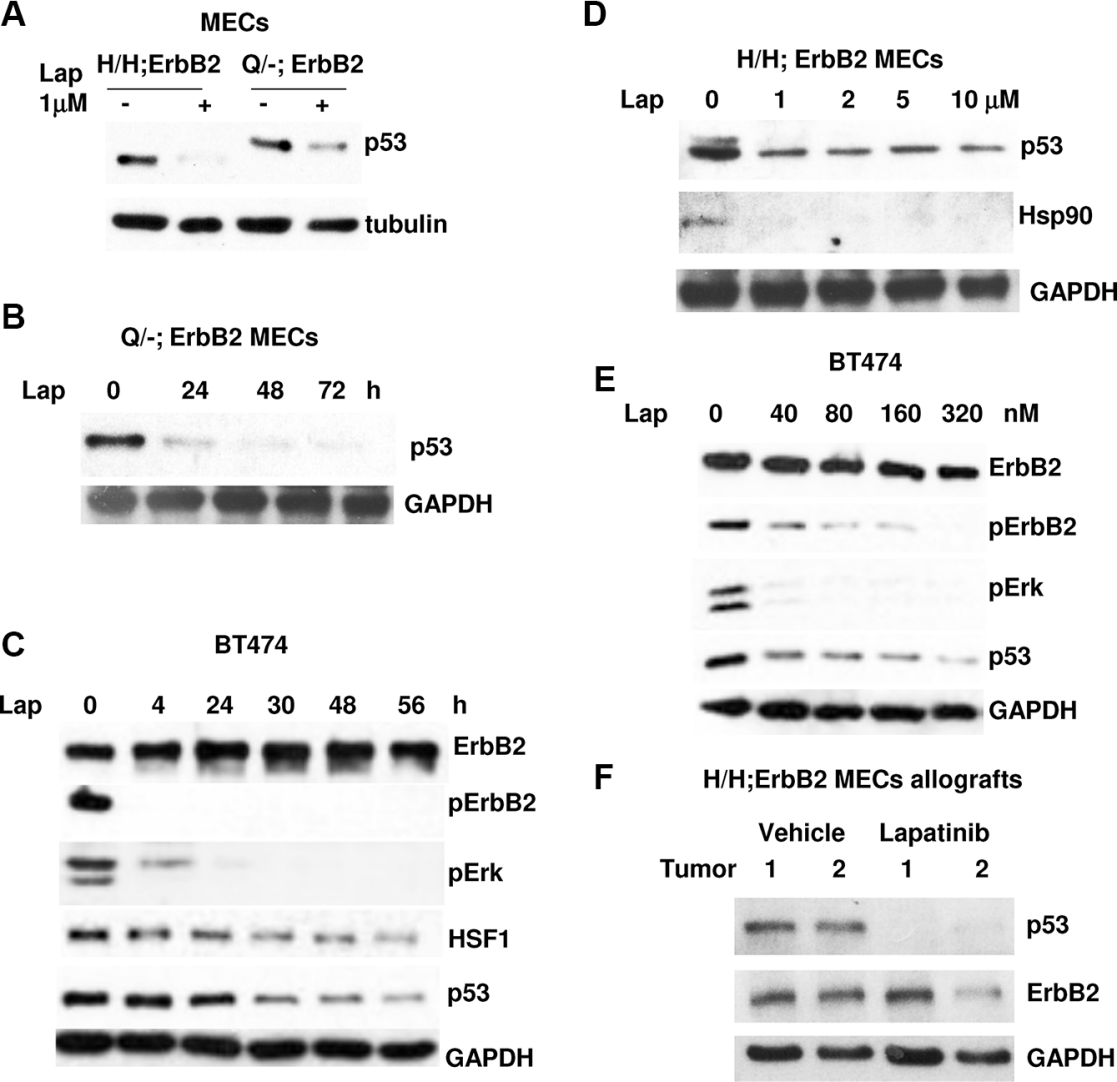
Lapatinib induces downregulation of mutp53 in ErbB2-expressing mammary cells (**A**) Lapatinib induces degradation of mup53 protein in H/H;ErbB2 and R248Q/-;ErbB2 MECs. Cells were treated with 1 μM of lapatinib for 24 h. (**B**) Mutp53 protein decline is detectable in 24 h after lapatinib treatment in murine Q/-;ErbB2 MECs. Cells were treated with 1 μM of lapatinib for indicated periods of times. (**C**) Mutp53 protein decrease is detectable in 24 h after lapatinib treatment in human BT474 cells. Cells were treated with 300 nM of lapatinib for indicated periods of times. (**D**) R172H mutp53 downregulation coincides with Hsp90 decline after lapatinib treatment. Cells were treated increasing concentrations of lapatinib for 48 h. (**E**) 40 nM of lapatinib is sufficient to block ErbB2 signaling, Erk phosphorylation and downregulate mutp53 in BT474 cells. Cells were treated increasing concentrations of lapatinib for 48 h. (**F**) 6–7 wks old Nu/Nu females (Harlan, strain Hsd:Athymic Nude-Foxn1nu) were subcutaneously injected into two dorsal sites with 2 × 10^6^ cells of cultured H/H;ErbB2 MECs per site. Mice were monitored twice weekly and upon appearance of palpable tumors were mock or lapatinib treated (100 mg/kg by oral gavage 3 times a week). At endpoint (tumor size ~3.5 cm^3^) in mock treated mice, animals were sacrificed. Tumors analyzed by Western blotting.

In support of our hypothesis, we found that inhibition of ErbB2 by lapatinib destabilizes mutp53, independently of type p53 mutation, in both H/H;ErbB2 and Q/-ErbB2 cultured mouse MECs (Figure 1A, 1B, 1D) and Her2 positive human breast cancer cell line BT474 (E285K) (Figure 1C, 1E). In murine and human cells mutp53 protein decrease is detectable in 24h after lapatinib treatment (Figure 1B, 1C). Importantly, the decline in pErbB2(Y1221/1222) and pErk levels (a hallmark of ErbB2 inhibition) was detectable as early as 4 h after lapatinib treatment and preceded mutp53 protein drop (Figure 1C). Furthermore, mutp53 downregulation coincides with both HSF1 (Figure 1C) and Hsp90 (Figure 1D) drop. The dose elevation experiment indicated that as low as 40nM of lapatinib is sufficient to block ErbB2 signaling, Erk phosphorylation and downregulate mutp53 (Figure 1E). *In vivo*, lapatinib suppresses tumor growth in allografted H/H;ErbB2 MECs, which was correlated with downregulation of mutp53 in tumors (Figure 1F). Together, our results imply that in addition to ErbB2/EGFR inhibition, lapatinib efficiently downregulates mutp53: i) *in vitro* and *in vivo*; *ii)* does not depend on type of p53 mutation (R172H vs R248Q mutation in murine cells with identical genetic background)*; iii)* does not depend on the cell host origin (human vs mouse).

### Lapatinib promotes degradation of mup53 protein

**Figure 2:**
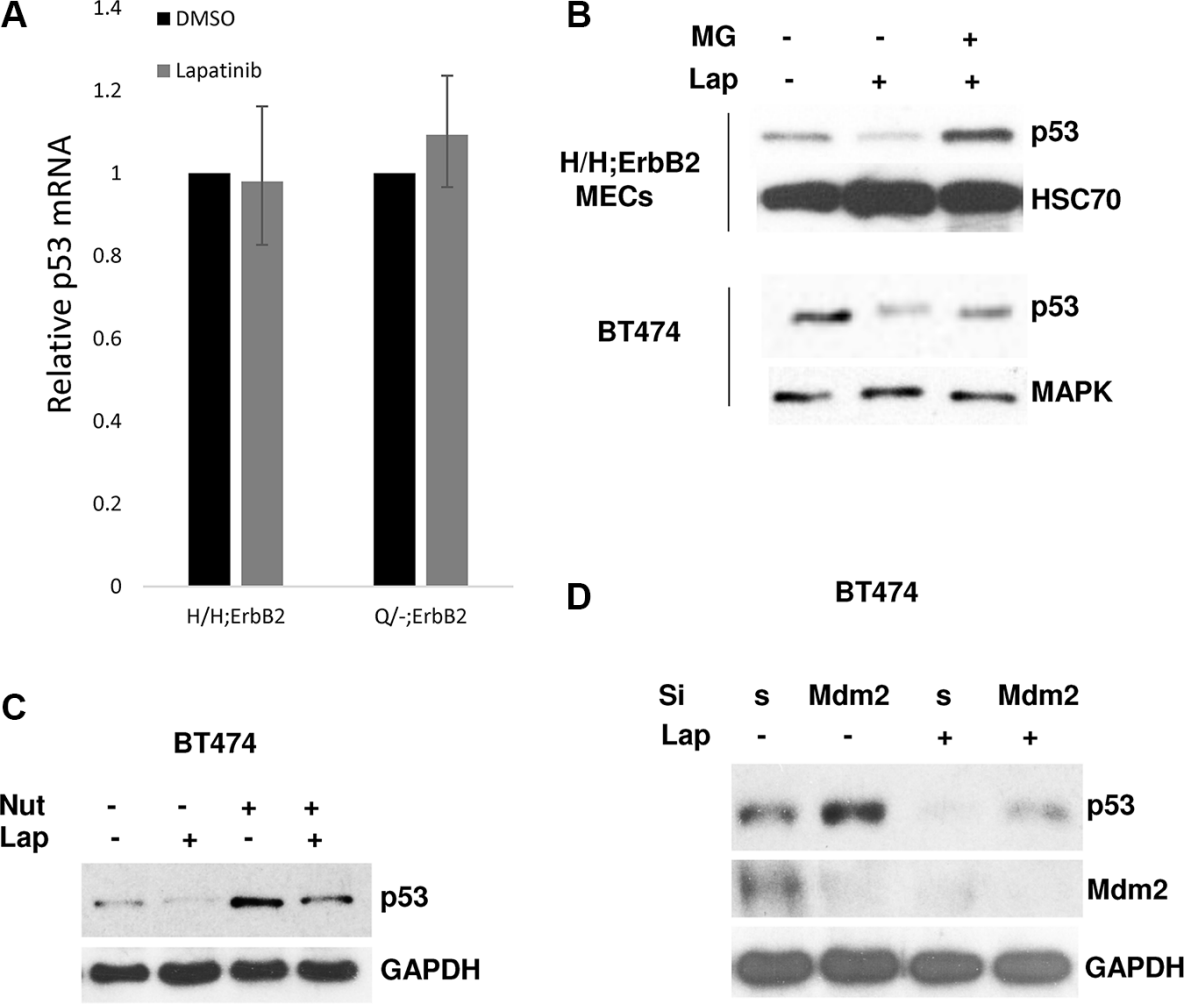
Lapatinib promotes degradation of mup53 protein (**A**) RNA levels were unchanged in both Q/-;ErbB2 and H/H;MECs before/after lapatinib treatment (1 μM for 48 h) p53 mRNA levels measured by qRT-PCR. Two independent experiments were performed in triplicate. (**B**) Proteasome inhibition by MG132 rescues lapatinib-mediated downregulation of mutp53 in H/H;ErbB2 MECs (top) and BT474 (bottom). Cells were simultaneously treated with lapatinib (300 nM) and MG132 (5 μM) for 24 h. (**C**) MDM2 inhibition by nutlin rescues lapatinib-mediated destabilization of mutp53. BT474 cells were simultaneously treated with lapatininb (300 nM) and nutlin (5 μM) for 24 h. (**D**) siRNA-mediated knockdown of MDM2 restores mutp53 levels after lapatinib treatment. BT474 cells were transfected with scrambled or siMDM2, followed by lapatinib treatment (300 nM) for 24 h. GAPDH as loading control.

To address the mechanism of lapatinib induced downregulation of mutp53, we tested whether lapatinib affects the transcription of mutp53 by quantitative RT-PCR analysis. We found RNA levels were unchanged in both Q/-;ErbB2 and H/H;MECs before/after lapatinib treatment (Figure 2A), suggesting that lapatinib downregulates mutp53 at post-transcriptional level.

Previously we [9] and others [20] have shown that in tumor cells aberrantly folded mutp53 proteins form stable complexes with Hsp90, which protects mutp53 from MDM2-mediated degradation. To test whether lapatinib induces degradation of mutp53 protein we treated mutp53 cells with proteasome inhibitor MG132. Indeed, proteasome inhibition by MG132 rescued lapatinib-mediated downregulation of mutp53 in both H/H;ErbB2 MECs and human BT474 cells (Figure 2B), confirming our notion that lapatinib promotes degradation of mutp53 protein. Previously, we and others have shown that E3 ligases Mdm2 as well as CHIP are inherently capable of degrading mutant p53 [8, 9]. Thus, specific MDM2 inhibitor nutlin blocks the interaction between MDM2 and mutp53 and stabilizes the latter (Figure 2C). Furthermore, both nutlin [23] (Figure 2C) and siRNA-mediated knockdown of MDM2 (Figure 2D) restore mutp53 levels after lapatinib treatment. Hence, our data implies that lapatinib induces degradation of mutp53 protein by re-activation of MDM2 E3 ligase activity. Previously, we have extensively studied the kinetics and activity of MDM2 in response to Hsp90 inhibition [9]. We found, in contrast to wtp53 harboring cells, MDM2 E3 ligase activity is selectively impaired in mutp53 expressing cells, while the physical interaction between endogenous mutp53 and MDM2 remains fully preserved [9]. Importantly, Hsp90 inhibition notably reduced the half-life of MDM2 and its bona fide substrates [9]. Thus, this study strongly supports the idea of enzymatic re-activation, self-ubiquitination and degradation of MDM2 in response to Hsp90 inhibition. As our model is based on the idea that ErbB2 signaling is upstream of Hsp90, we believe that a similar mechanism of MDM2 destabilization takes place upon ErbB2 inhibition. In Figure 2D we show that lapatinib decreases MDM2 level, which can be explained by enhanced MDM2 E3 ligase activity, its autoubiquitination and degradation (Figure 2D, compare lanes 1 and 3). Together, our data indicates that lapatinib restores MDM2 activity, followed by both mutp53 protein degradation, MDM2 autoubiquitynation and self-degradation.

### Lapatinib destabilizes mutp53 via modulation of HSF1 activity

**Figure 3:**
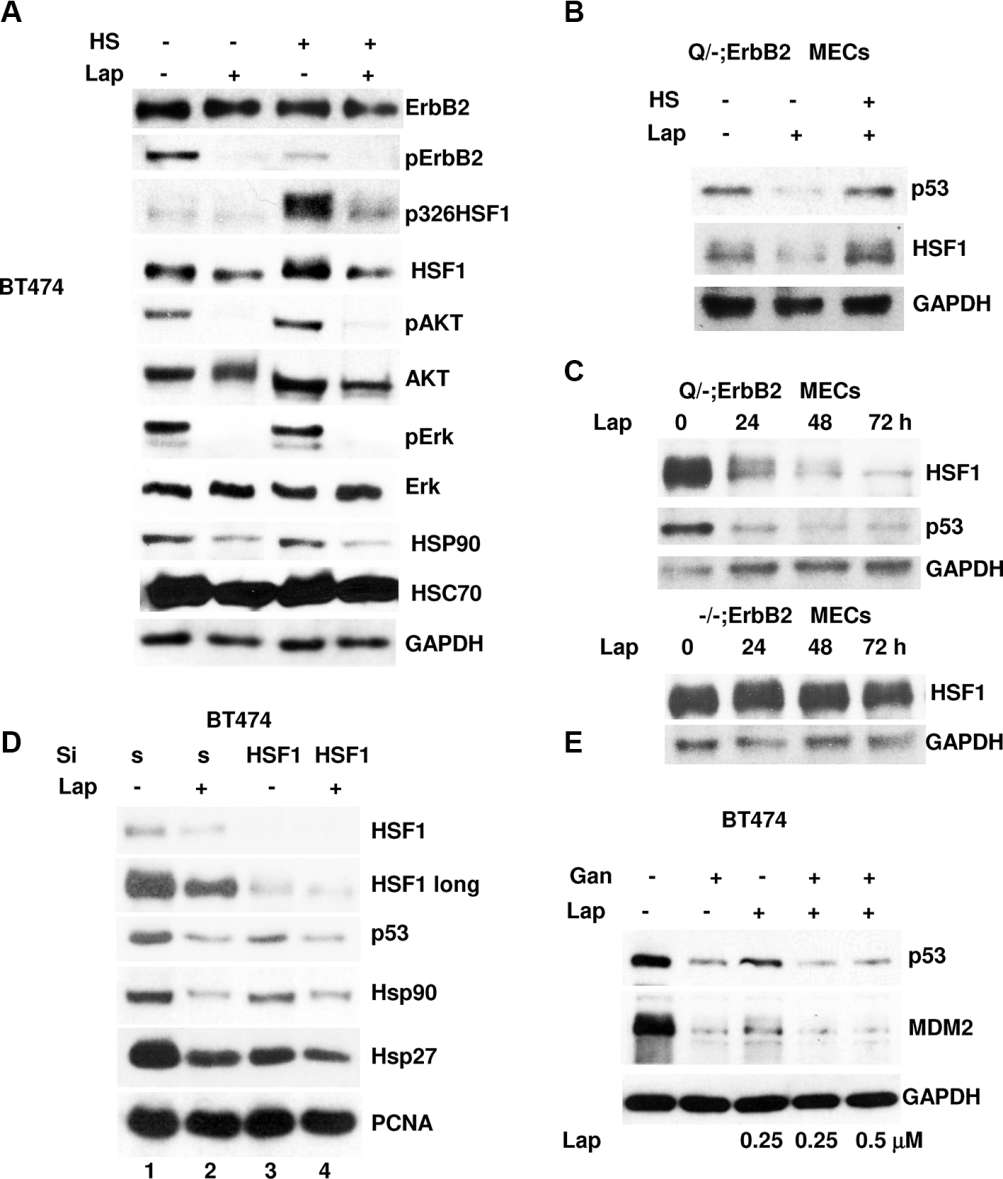
Lapatinib destabilizes mutp53 via modulation of HSF1 activity (**A**) Lapatinib inhibits HSF1Ser326 phosphorylation induced by heat shock in BT474 cells. Cells were pre-treated with 300nM of lapatinib for 24 h. After heat shock (42°C, 30 min) cells were immediately analyzed by immunoblot. (**B**) Heat shock (42°C, 30 min) alleviates mutp53 destabilization by lapatinib in p53Q/-;ErbB2 MECs. Cells were pre-treated with lapatinib (300 nM, 24 h). After heat shock (42°C, 30 min) cells were immediately analyzed by immunoblot. (**C**) Lapatinib (300 nM) mediated mutp53 destabilization coincides with reduction of HSF1 levels in Q/-;ErbB2, but not in p53−/−;ErbB2 MECs. (**D**) In HSF1-ablated BT474 cells, lapatinib (300 nM) does not induce further destabilization of mutp53. Cells were transfected with siHSF1 or scrambled siRNA control. After 24 h, cells were treated with 300 nM of lapatinib for an additional 24 h followed by immunoblot. (**E**) Lapatinib destabilizes mutp53 only in the presence of functional Hsp90. BT474cells were pre-treated with ganetespib (250 nM) for 6 h followed by lapatinib (250 nM and 500 nM) treatment for 24 h. Lapatinib even at high concentrations does not further decrease mutp53 and MDM2 levels in cells pre-treated with Hsp90 inhibitor.

Our previous studies identified HSF1 and its transcriptional target Hsp90 as important determinants of mutp53 stability [19]. Furthermore, we found that ErbB2 and/or EGFR signaling via phosphorylation HSF1 at Ser326 induces transcriptional activation of HSF1 [19], which also protects HSF1 from polyubiquitination and proteasomal degradation [24]. Importantly, lapatinib blocks phosphorylation of downstream effectors of ErbB2 - AKT and Erk, which has been shown to play an important role in transcriptional activation of HSF1 by Ser326 phosphorylation [24, 25] (Figure 3A). Consistent with these data, we found that lapatinib blocks AKT, Erk and Ser326 HSF1 phosphorylation induced by heat shock (42°C, 30 min) in BT474 cells (Figure 3A). Furthermore, lapatinib downregulates HSF1 levels concomitant with the Hsp90 drop, but does not affect constitutive HSP- Hsc70 (Figure 3A). Also, transcriptional activation of HSF1 by heat shock alleviates lapatinib induced mutp53 degradation in p53Q/-;ErbB2 MECs (Figure 3B). Seemingly, lapatinib affects HSF1 signaling in mutp53-dependent, since HSF1 drop after lapatinib treatment occurs only in mutp53 expressing (Q/-;ErbB2), but not in p53 −/−;ErbB2 MECs (Figure 3C). To further prove that lapatinib destabilizes mutp53 via modulation of HSF1 activity, we examined the effect of HSF1 silencing. As expected, we found that siRNA mediated HSF1 ablation downregulates mutp53 (Figure 3D, compare lanes 1 and 3). Nevertheless, lapatinib does not induce further destabilization of mutp53 in the absence of HSF1 (Figure 3D, compare lanes 2 and 4). Consistent with mutp53 as a Hsp90 client, Hsp90 downregulation after HSF1 ablation was concomitant with mutp53 decline (Figure 3D). To further prove that lapatinib destabilizes mutp53 via HSF1 transcriptional target Hsp90, we pre-treated BT474 with Hsp90 inhibitor ganetespib [18] for 6h followed by lapatinib treatment for 24 h. We found that lapatinib, even at high concentrations, does not further decrease mutp53 and MDM2 levels in cells pre-treated with ganetespib (Figure 3E). These results suggest that lapatinib could target mutp53 for degradation only in the presence of functional Hsp90.

In sum, these experiments support our hypothesis that inhibition of ErbB2 by lapatinib suppresses HSF1 transcriptional activation (Figure 3A) and protein levels (Figure 1C) with subsequent decline of its target Hsp90, releasing mutp53 from the Hsp90 inhibitory complex followed by MDM2 reactivation and mutp53 degradation.

### Mutant p53 sensitizes cells to lapatinib

The therapeutic benefit of targeting mutp53 was established by Alexandrova et al. in recent proof-of-principle experiments. They have shown that the genetic deletion of mutp53 from T/B cell lymphoma tumors inhibits their growth and extends survival of mutp53 knock-in mice [18]. Thus, in addition to ErbB2 inhibition, lapatinib-induced destabilization of mutp53 protein could potentiate its therapeutic effect specifically in mutp53 harboring tumors. Hence, we hypothesized that mutp53 allele may sensitize ErbB2 expressing cells to lapatinib by two complementary mechanisms: 1) mutp53 mediated amplification of ErbB2 signaling [17] that creates superior dependency of cancer cells on ErbB2 signaling, and 2) lapatinib induced mutp53 degradation (Figures 1, 2). Therefore, mutp53 harboring cells could be more responsive to ErbB2 inhibition than p53null counterparts and, possibly, wtp53 cells.

**Figure 4:**
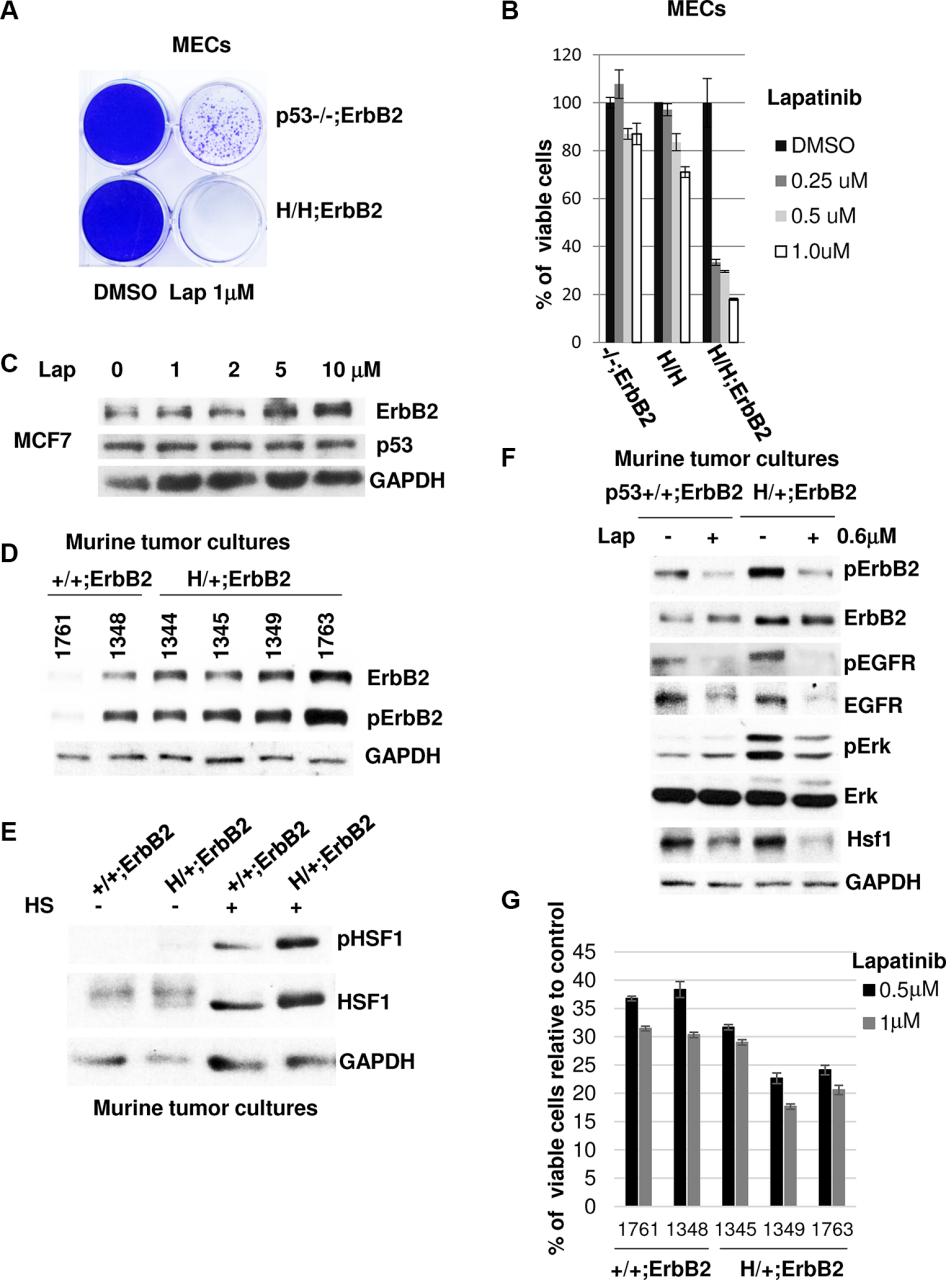
Mutant p53 sensitizes cells to lapatinib (**A**) Lapatinib shows preferential cytotoxicity in mutp53;ErbB2 mammary cells. H/H;ErbB2 MECs show higher sensitivity to lapatinib, compared to p53−/−;ErbB2 counterparts in colony formation assay (crystal violet staining). H/H;ErbB2 and −/−;ErbB2 MECs were treated with 1 μM of lapatinib for 5 weeks. (**B**) H/H;ErbB2 MECs show higher viability loss in response to lapatinib compered to −/−;ErbB2 and H/H MECs. Cells were treated with indicated concentrations of lapatinib for 48h. Viability loss is shown relative to DMSO treated controls. (**C**) Lapatinib destabilizes mutp53 but not wtp53. Lapatinib does not affect wild-type p53 protein even at high concentrations. MCF7 cells were treated with indicated concentrations of lapatinib for 24 h. (**D**) Mammary tumor cell lines show both elevated ErbB2 and pErbB2 levels (Y1221/1222) in the presence of the mutp53 allele compared with p53 +/+;ErbB2. Cells were established from primary mammary tumors of mice with indicated genotypes. (**E**) Heat shock (42°C, 30 min) more potently induces Ser326HSF1 activation in the presence of mutp53 allele compared with p53+/+;ErbB2 tumor cell lines. After heat shock p53+/+;ErbB2 (#1761) and H/+;ErbB2 (#1349) cells were immediately analyzed by immunoblot. (**F**) Lapatinib more profoundly inhibits of ErbB2 downstream signaling (pErk, pErbB2, HSF1) in H/+;ErbB2 compared with p53+/+;ErbB2 cancer cells. Cells were treated with lapatinib (0.6 μM, 24 h) and analyzed by immunoblot. (**G**) Tumor cell lines harboring mutp53 allele respond better to lapatinib measured by cell viability assay. Mammary tumor cell lines with indicated genotypes were treated with 0.5 and 1 μM of lapatinib for 48 h. Viability loss is shown relative to DMSO treated controls (calculated as 100% viability).

**Figure 5:**
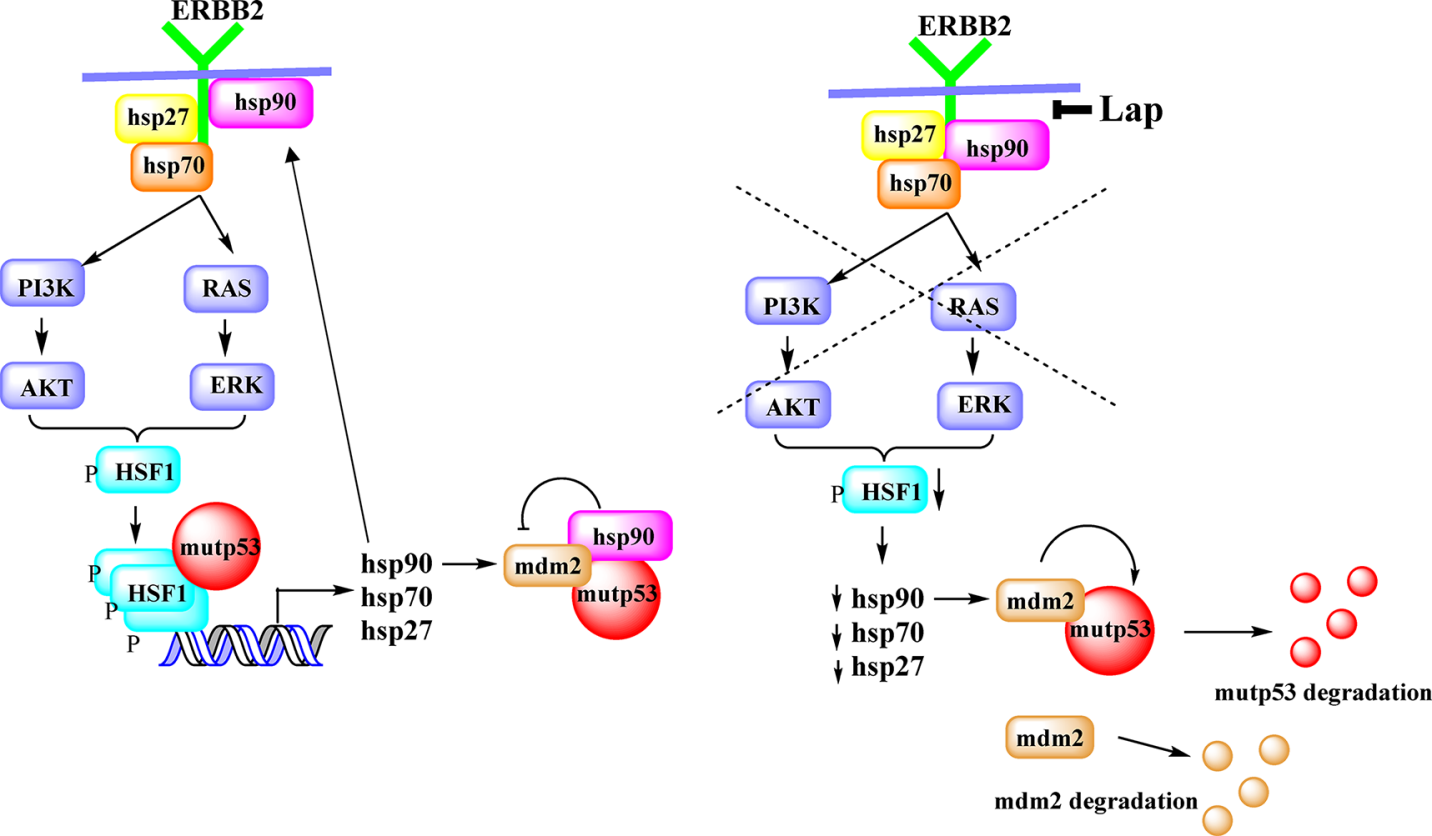
Proposed model ErbB2 signaling mediates HSF1 activation in a mutp53-dependent manner. Mutp53, by enhancing ErbB2 signaling, potentiates HSF1 activity via a feed-forward loop and thereby upregulates Hsp90 clients, including mutp53. Inhibition of ErbB2 by lapatinib, leads to inhibition of HSF1 transcriptional function, decline in Hsp90 level, release MDM2 from inhibitory complex and subsequent degradation of mutp53 and MDM2.

Indeed, we found that H/H;ErbB2 MECs are more sensitive to lapatinib than their p53−/−;ErbB2 counterparts in both a colony formation assay (Figure 4A) and a cell viability assay (Figure 4B). In agreement with our model (Figure 5), mutp53 sensitizes MECs to lapatinib only in the presence of ErbB2 but not in MECs established from H/H knock-in mice (Figure 4B).

Although this data strongly supports the notion of the oncogenic cooperation of mutp53 and ErbB2, it has limited clinical application. Contrary to p53 mutations, wtp53 deletions are rather rare in breast cancer. Hence, the comparison of wtp53;ErbB2 and mutp53;ErbB2 cancer cells more faithfully recapitulates human ErbB2 positive breast cancer. Even though our previous studies identified novel oncogenic activity of mutp53 in amplification of ErbB2 signaling [17], it is not clear how wtp53 status impacts ErbB2 signaling and the response to ErbB2 targeted therapies. Contrary to mutp53, we did not observe wtp53 protein decline in response to Hsp90 inhibition in our previous study [9]. Consistently, lapatinib even at high doses does not affect wtp53 level in human MCF7 breast cancer cell line. Although MCF7 cell line is derived from ER-positive human breast cancer, MCF7 cells show detectable levels of ErbB2 (Figure 4C). Since only limited amount of wtp53;ErbB2 human cell lines are available for analysis, we established cell lines from mammary tumors of littermates p53 +/+;ErbB2 vs H/+;ErbB2 mice. In contrast to human cell lines, the identical genetic background of these mice helps to delineate mutp53-specific effects in ErbB2 context and the response to ErbB2 targeted therapies in a well-controlled experimental setting.

Consistent with our previous findings [17], we detected both elevated ErbB2 and pErbB2 levels in the presence of the mutp53 allele compared with p53 +/+;ErbB2 mammary tumor cell lines (Figure 4D). In further support of ErbB2 as an upstream effector of HSF1 activation, heat shock (42°C, 30 min) more potently induces Ser326HSF1 activation in the presence of mutp53 allele compared with p53+/+;ErbB2 tumor cell lines (Figure 4E). As a result of mutp53-mediated enhancement of ErbB2 signaling, lapatinib shows stronger inhibition of EGFR and ErbB2 signaling in the presence of mutp53 allele (compare ratio of pErbB2 and pEGFR in mock and lapatinib treated cells) (Figure 4F). Consequently, the inhibition of downstream Erk signaling is more pronounced in H/+;ErbB2 compared with p53+/+;ErbB2 cancer cells. In accord with our earlier findings (Figure 3), higher efficiency of ErbB2/EGFR inhibition in mutp53 harboring cells coincides with more robust decline of HSF1 levels (Figure 4F). Hence, compared to wtp53 cells, enhanced ErbB2 signaling in mutp53 harboring cells could generate higher addiction to ErbB2 pathway. Indeed, we found better response to lapatinib in H/+;ErbB2 tumor cell lines in H/+;ErbB2 compared to p53+/+;ErbB2 cancer cells measured by cell viability assay (Figure 4G). These results are strongly supported by meta-analysis of the COSMIC drug sensitivity database of 226 human cancer cell lines (representing breast cancer as well as other cancer types) ( http://www.cancerrxgene.org/translation/Drug/119). Specifically, we found that mutp53 human cell lines are more sensitive to lapatinib than wtp53 cells (*p* = 0.0408).

Together, our data implies that in comparison to wtp53 and p53 null cancer, mutp53–mediated amplification of ErbB2 function could generate superior addiction of cancer cells to ErbB2 signaling. Thus, mutational status of p53 could serve as a potential predictive biomarker for better clinical response to ErbB2 targeted therapies in breast cancer cells.

## DISCUSSION

ErbB2/Her2, a member of the human epidermal growth factor receptor family, is highly overexpressed in 20–30% of all breast cancer cases [3]. High levels of ErbB2 in cancer cells induce ligand-independent constitutive dimerization of ErbB2 and/or dimerization with other epidermal growth factor receptor family members, triggering downstream signaling through the phosphoinositide-3-kinase (PI3K)–AKT and Ras–Raf–MEK–ERK1/2 cascades [3]. Activation of these signaling pathways promotes cell proliferation and invasion, thus, enabling cancer progression and metastases. And while development of Her2-targeted therapies significantly improves patient outcomes, the primary and acquired resistance to these modalities remain a major clinical concern. Therefore, our understanding of how ErbB2 cooperates with other oncogenic pathways in context of ErbB2 targeted therapies is critical for improvement of therapeutic outcomes in these high risk breast cancer patients.

Our previous *in vivo* studies found strong evidence of oncogenic cooperation of mutp53 and ErbB2. By crossing mutp53 R172 knock-in mice with ErbB2/Neu transgenic mice we discovered that the mutp53 R172H allele is a more potent activator of ErbB2 mammary tumorigenesis than simple loss of p53, reflected by more aggressive disease, earlier tumor onset, increased tumor multiplicity and shorter survival [17]. These findings are in agreement with clinical data that mutations in the p53 gene are the most frequent oncogenic events in Her2 positive breast cancer [1], which are highly predictive of poor disease outcome [4]. Despite of evident negative impact of mutp53 on ErbB2 breast cancer development, p53 mutational status is not routinely used as a guide for therapy planning in breast cancer. In this study we evaluated potential predictive value of mutational p53 status in response to Her2 targeted therapies.

We and others have previously shown that high mutp53 protein levels in cancer cells depend on heat shock protein Hsp90 [9, 18, 20]. Although basal Hsp90 protein level is highly abundant in cancer cells, it is further transcriptionally induced in response to environmental stress. It has been shown that eukaryotic cells express both constitutive Hsp90β and stress-inducible cytosolic Hsp90α. It is well established that stress-induced transcription of Hsp90α is controlled by the transcription factor HSF1 [26].

As a transcription factor, HSF1 controls a broad spectrum of events essential for protecting cells from proteotoxic stress, which is associated with the accumulation of misfolded proteins, e.g. in cancer cells. Thus, HSF1 activates transcription of genes that regulate protein homeostasis, including the molecular chaperones Hsp27, Hsp70, Hsp90 [26]. Unlike normal cells, tumor cells are characterized by a permanently high rate of protein misfolding due to abundance of mutated oncoproteins, making HSF1 ubiquitously and constitutively overexpressed [26]. Hence, HSF1 protein levels are elevated in 80% of breast cancer, leading to enhanced expression of its targets, including Hsp90 [27]. Most importantly, HSF1 transcriptional targets Hsp90 [28, 29], Hsp70 [28] and Hsp27 [26] are responsible for ErbB2 protein stability. The critical significance of HSF1-regulated heat chock response in ErbB2 mediated mammary tumorigenesis was proven by *in vivo* genetic model. Genetic knockout of HSF1 suppresses mammary hyperplasia and reduces tumorigenesis in ErbB2 transgenic mice *in vivo* [30]. Meanwhile, oncogenicity of mutp53 also critically depends on HSF1 function. In the absence of HSF1, mutp53 H/+ KI mice show a 70% reduction in tumor formation [31].

To explore potential mutp53-HSF1-ErbB2 link, we recently performed a series of mechanistic studies and described a novel mutp53-initiated oncogenic feed-forward loop, which governs resistance of cancer cells to proteotoxic stress that enables cancer cells superior survival [17]. We propose the model whereby mutp53 through enhanced recycling (similar to EGFR [13]) and/or stability of ErbB2 [17], augments MAPK and AKT signaling leading to transcriptional phospho-activation of HSF1 at Ser326 [24, 25]. Furthermore, we established that mutp53 directly interacts with phospho-activated HSF1 and facilitates its binding to DNA response elements, thereby stimulating transcription of HSPs. In turn, HSPs more potently stabilize their clients ErbB2, EGFR, mutp53, HSF1 (and possibly other oncogenes), thus, reinforcing tumor development (Figure 5) [17]. Consistently, we found that ErbB2 inhibition by lapatinib not only strongly suppressed tumor progression in ErbB2 mice, but does so, at least in part, via *inactivation of HSF1* [32]. In agreement, in present study we found that targeting of ErbB2 by lapatinib inhibits phospho-activation of HSF1 at Ser326 and destabilizes HSF1 protein (Figure 3A). Together with previous findings that ErbB2-driven mammary tumorigenesis is suppressed in HSF1 knockout mice [30], our results strongly support the proposed model (Figure 5). Furthermore, relevant to human disease, we found a strong correlation between mutp53 and nuclear p-Ser326 HSF1 in 150 human breast cancer biopsies (by immunochemistry) *only in Her2-positive tumors*. No correlation between mutp53 and pSer326-HSF1 staining was found in Her2-negative;ER/PR-positive breast cancer samples [19]. Altogether, this data provides a mechanistic explanation of how mutp53 potentiates ErbB2 signaling and modulates the response to ErbB2 targeting compounds (Figure 5).

Importantly, discovery of oncogenic function of mutp53 in the upregulation of heat shock response and ErbB2 signaling, opens up novel therapeutic opportunities. We found that *higher dependency* on the ErbB2-HSF1-mutp53 loop sensitizes mutp53;ErbB2 cancer cells to the interception of any of its components. The interference of proposed feed-forward loop by lapatinib inhibits HSF1 function (Figure 3), followed by Hsp90 decline and MDM2-mediated mutp53 degradation (Figure 2). Therapeutic benefit of targeting mutp53 was previously established in various cancer models [11, 18]. Hence, mutp53 status in ErbB2 positive cancer cells predicts higher sensitivity to lapatinib via two complementary mechanisms: mutp53-mediated amplification of ErbB2, and simultaneous targeting of potent oncogenic drivers, ErbB2, mutp53 and HSF1 by ErbB2 inhibition. Indeed, our *in vitro* and *in silico* analysis confirms this hypothesis and shows superior response to lapatinib in mutp53 harboring cells compared with p53 null and wtp53 cancer cells (Figure 4).

Many Her2-targeted drugs are currently on the market (trastuzumab, pertuzumab, lapatinib, TDM-1) or have recently entered clinical trials, e.g. CI-1033 (Pfizer), NVP-AEW541 (Novartis) and Perifosine (Keryx) [33]. In this study, we primarily focused on the small molecule inhibitor lapatinib, since human Her2-specific monoclonal antibodies trastuzumab and pertuzumab cannot be studied in mouse models. However, our data strongly suggest that mutp53 harboring breast cancer cells also could be sensitized to Her2-antibody based therapies via similar mechanism. This important clinical question should be addressed in further retrospective human clinical studies.

Overall, our data provides important information that can help to improve treatment options for ErbB2-positive breast cancer patients. We showed that pharmacological targeting of ErbB2 leads to destabilization of mutp53 protein via modulation of heat shock response, and therefore, could be more therapeutically beneficial specifically for mutp53 harboring patients.

## MATERIALS AND METHODS

### Human cancer cells

Human Her2 positive breast cancer cell line BT474 (E285K p53 mutation) and human breast cancer cell line MCF7 (contain functional wtp53) were obtained from ATCC. Where indicated, cells were treated with indicated concentrations of lapatinib (LC lab, # L-4899). Concentrations of lapatinib were optimized for every experimental setting, depending on cell types. Where indicated cells were treated with 5 μM MG132 (Sigma) and 5 μM nutlin (Sigma) added to the medium. All cell viability assays were done using standard clonogenicity assays and CellTiter-Blue Cell Viability Assay (Promega, 96-well format with 5,000 cells/well seeded 24 hrs prior). Cells were treated for 48 hours in various concentrations of drug used. Florescence was detected by SPECTRAmax M2 (Molecular Devices).

### RNA interference

Pools of 4 different siRNA duplexes specific for human HSF1 (Dharmacon), MDM2 (Ambion) or scrambled control duplexes were transfected with Lipofectamine 2000 (Invitrogen). Cells were harvested 48 h later for analysis.

### Immunoblots

For immunoblots, equal total protein of cell lysates (2.5–20 μg) were detected with antibodies to mouse p53 (FL393), human p53 (PAb1801), MDM2, GAPDH, HSC 70 (all Santa Cruz Biotechnology), Erk, pErk (T202/Y204), EGFR, EGRF-Tyr845P, ErbB2, pErbB2 (Y1221/1222) (all Cell Signaling), HSF1, pSer326 HSF1, Hsp90 (Enzo Life Sciences, Inc., Farmingdale, NY).

### Quantitative PCR

Total RNA from cells was isolated using Trizol (Invitrogen) according to manufacturer's guidelines. Equal amounts of RNA were reverse-transcribed and real-time PCR analysis was performed using qPCR Master-Mix (75 mM Tris-HCl, pH 8.8, 20 mM (NH4)2SO4, 0.01% Tween-20, 3 mM MgCl2, SYBR Green 1:80,000, 0.2 mM dNTPs, 20 U/ml Taq-polymerase, 0.25% TritonX-100, 0.3M Trehalose and 0.3 mM primers).

### Mice

MMTV-ErbB2 mice harboring activated ErbB2 (strain FVBN-Tg(MMTV-ErbB2)NK1Mul/J) were from Jackson Labs. p53 R172H (called p53H/H) and control p53 null (p53−/−) mice (C57Bl6J background) were a gift from G. Lozano [34] . Humanized R248Q knock-in mice were a gift from Dr. Moll [18], knock-in p53 mice were interbred to generate H/- and Q/- mice. Compound p53H/-;ErbB2 and Q/-;ErbB2 mice were generated by crossing ErbB2 into the p53−/− background and then breeding the p53+/−;ErbB2 progeny with p53H/H and p53 Q/Q animals. H/-;ErbB2 mice were then crossed to generate p53H/H;ErbB2 and p53−/−;ErbB2 females for analysis. These F2 mice were of mixed background. Littermates were used for all analyses. Mice were regularly monitored and euthanized when they became moribund. Careful necropsies were performed and tumors and all major organs collected. Mice were treated according to guidelines approved by the Institutional Animal Care and Use Committee.

### Allografts

6–7 wks old Nu/Nu females (Harlan, strain Hsd:Athymic Nude-Foxn1^nu^) were subcutaneously injected into two dorsal sites with 2 × 10^6^ cells of cultured H/H;ErbB2 MECs per site suspended in 3:1 PBS/Matrigel (BD Biosciences). Mice were monitored twice weekly and upon appearance of palpable tumors were mock (18% Cremophor/3.6% dextrose) or lapatinib treated (100 mg/kg by oral gavage 3 times a week). At endpoint (tumor size ~3.5 cm^3^) in mock treated mice, mice were sacrificed. Tumors were analyzed by Western blotting.

### Mammary cells cultures

Mammary glands were dissected from 8 wk-old virgin female mice and sequentially digested at 37°C for 2 h in collagenase/hyaluronidase, 0.05% Trypsin, DNAse I and Dispase (Stem Cell Technology). The ensuing cell suspension was treated with red blood cell lysis buffer, rinsed with PBS, and passed through a 40 μm mesh after resuspension in Opti-Mem medium (Gibco). Cells were plated on gelatin-coated plates and grown in CnT-BM1 medium (Cell-N-Tec). For the establishing mammary tumors culture, mammary tumors were dissected, rinsed three times in PBS, minced and processed as described above.
